# Increasing the Accuracy of Hourly Multi-Output Solar Power Forecast with Physics-Informed Machine Learning

**DOI:** 10.3390/s22030749

**Published:** 2022-01-19

**Authors:** Daniel Vázquez Pombo, Henrik W. Bindner, Sergiu Viorel Spataru, Poul Ejnar Sørensen, Peder Bacher

**Affiliations:** 1 Department of Electrical Engineering, Technical University of Denmark (DTU), 4000 Roskilde, Denmark; hwbi@elektro.dtu.dk; 2 Research and Development, Vattenfall AB, 169 56 Solna, Sweden; 3 Photovoltaic Materials and Systems, Technical University of Denmark (DTU), 2800 Kgs. Lyngby, Denmark; sersp@fotonik.dtu.dk; 4 Department of Wind Energy, Technical University of Denmark (DTU), 4000 Roskilde, Denmark; posq@dtu.dk; 5 Department of Computer Science, Technical University of Denmark (DTU), 2800 Kgs. Lyngby, Denmark; pbac@dtu.dk

**Keywords:** solar power forecasting, deep-learning, physics-informed machine learning, PV

## Abstract

Machine Learning (ML)-based methods have been identified as capable of providing up to one day ahead Photovoltaic (PV) power forecasts. In this research, we introduce a generic physical model of a PV system into ML predictors to forecast from one to three days ahead. The only requirement is a basic dataset including power, wind speed and air temperature measurements. Then, these are recombined into physics informed metrics able to capture the operational point of the PV. In this way, the models learn about the physical relationships of the different features, effectively easing training. In order to generalise the results, we also present a methodology evaluating this physics informed approach. We present a study-case of a PV system in Denmark to validate our claims by extensively evaluating five different ML methods: Random Forest, Support Vector Machine, Convolutional Neural Networks (CNN), Long-Short Term Memory (LSTM) and a hybrid CNN–LSTM. The results show consistently how the best predictors use the proposed physics-informed features disregarding the particular ML-method, and forecasting horizon. However, also, how there is a threshold regarding the number of previous samples to be included that appears as a convex function.

## 1. Introduction

One of the fastest growing renewable energy sources is solar power due to its cost effectiveness, deployment simplicity and low maintenance costs. Particularly, Photovoltaic (PV) technology is the solar generation technology most widely deployed, representing well above 90% of the installed power [1]. The inherent stochasticity of the solar resource challenges the operation of the generation units, particularly regarding the estimation of future available power as it depends on latitude, season, humidity, cloud conditions, air quality, pollution, etc. which are only captured by local estimators [2]. Different methods and technologies have been developed as to allow the integration of ever-growing rates of renewable energy such as demand response, energy storage, etc. Out of them, forecasting is proven to be the most economical and simple method to accommodate higher shares of renewable in the power system without compromising safety [3]. However, solar forecasts are usually focused on irradiance prediction which has the advantage to be general for any solar power technology, but ignores that, from an energy management perspective, the metric of interest is the actual power. This requires to post-process the forecasted irradiance as to estimate power; ultimately yielding low accuracy when compared to direct PV power prediction [2]. These methods employ a black-box approach, not allowing a transparent interpretation of the processed variables affecting the accuracy of the model [4]. On the other hand, by predicting power directly, the forecast model is, by definition, tailor-made for a specific installation.

In general, Machine Learning (ML) based methods use pattern recognition capabilities of artificial intelligence to perform regression operations. Lately, there has been an increase in the number of publications related to ML-based solar forecasting due to its conceptual simplicity, widely available datasets and excellent results, however, focusing more on irradiance rather than power [5]. The most accurate methods for solar forecasting as identified from the available literature are: Random Forest (RF), Support Vector Regression (SVR), Long-Short Term Memory (LSTM), Convolutional Neural Networks (CNN), and a hybrid CNN–LSTM [2,6,7]. We also include persistence, as it is a common benchmark method widely employed in the literature, and Semi-Parametric Auto-Regressive (SPAR) since it represents a non-naive forecasting method with far stronger prediction capabilities [8]. From the point of view of the training data inclusion, models can be divided in those using solely intrinsic data (the predicted variable, active power in our case), and others including additional extrinsic or exogenous features that could provide additional information. Intrinsic models are simpler and faster to train, however, exogenous hold the potential of being more accurate due to the additional information. That is, learning linear and non-linear relationships between the features. Typical, exogenous features are, meteorological recordings, Numerical Weather Predictions (NWP), and satellite or sky images [2].

There is a lot of published work regarding hourly solar forecasting with ML focusing on either one or few steps ahead up to 24 (one day-ahead) [2,9,10]. Particularly, it is possible to see a shift from intrinsic models, towards exogenous, where the most common approach is to include meteorological features. In more recent times, imaging has been included to improve forecasts up to 6 h ahead, while NWP has proven to improve accuracy for day-ahead horizons [11]. However, most of the available work focuses on direct irradiance forecasting and power modeling. For example, in [12] hourly irradiance values are fed to a SVR to forecast few steps ahead of Global Horizontal Irradiance (GHI); while in [13,14] meteorological recordings are fed to an RF and CNN, respectively, to predict irradiance. In [15], recordings from neighbouring sites are used in their forecast given their correlation. Lastly, [16] proposes an ensemble method training three Artificial Neural Networks (ANN) where one uses meteorological recordings, another NWP and the last one casts 24 h-ahead predictions based on the outputs of the first two.

Clearly, there are still unaddressed issues in the aforementioned researches, which can also be identified from several different reviews [2,6,7,9,10]. For instance, there is very little work focused on direct power forecasting, also, most publications focus on a single ML-model, thus rarely assessing them against others. In fact, the lack of comprehensive comparisons between ML-models has been identified as a gap in several recent reviews [2,10]. In addition, introducing physics-informed features in ML-models is a rising trend in different disciplines. Despite the promising results that have been reported for meteorological applications, there is virtually no work covering such topic for PV power forecasting [17]. In this direction, we propose the inclusion of a PV-performance model as part of the data-mining stage. In this way, we effectively include knowledge of the physical relationships between meteorology and an array’s operational state. This model only requires wind speed and air temperature measurements, along with some basic data from the PV’s datasheet, thus making it extremely easy to implement in practice. Then, given the difficulties in generalising performance in ML applications, we propose a methodology aiming to find performance patterns across the different ML-models via systematic comparison of five different methods: RF, SVM, CNN, LSTM and CNN–LSTM; but also SPAR and a naive Persistence. We evaluate the proposed physics-informed approach in a study-case with a PV array installed in Denmark for three different horizons: 24, 48 and 72 h-ahead. These horizons were selected based on their relevance in classic market participation, energy management applications and on the general gap pointing towards the lack of multi-output predictors beyond six steps head [18]. Lastly, we recommend a feature selection and tuning approach based in physics-informed criteria.

This work extends previous studies, which were either focused on forecasting solar irradiance [14], or their feature selection was solely based on correlation [19]. Others applied seemingly arbitrary criteria when deciding between intrinsic vs. extrinsic feature inclusion [20], or how to effectively select informative features [21]. The results show how the different models are quite consistent regarding the most suitable feature combinations and number of previous samples to be included, how Pearson correlation failed to identify useful features and how different ML-methods are better suited for different horizons. Lastly, it is worth mentioning that we have followed the recommendation of [22], which sharply criticises the bias in solar forecasting regarding the systematic non-publication of under-performing methods. Therefore, besides showing the best performers, we analyse each individual method. The main contributions of this paper can be summarized as follows:

- The adaptation of a PV-performance model as to include it in any ML-model, which allows to add physics-informed features as a recombination of basic meteorological recordings and manufacturing parameters available at the PV’s data-sheet. These hold information regarding the operational state of the array, effectively easing training and allowing to obtain higher accuracy.
- Proposal of a methodology suitable to extend the findings of a study-case to a generalization, this is achieved by recurrent grid search of different combinations of: features, number of previous samples and hyperparameters.
- Proposal of a physics-informed feature selection. This allows to discard redundant features, effectively reducing the search-space without compromising the location of the best forecaster. In addition, it also permits to keep features keeping non-linear relationships that would be removed from the dataset given their low correlation.
- Extensive systematic comparison of ML-methods: RF, SVM, CNN, LSTM and CNN–LSTM applied to hourly PV power forecast based solely on historical data for three different horizons: 24, 48 and 72 h ahead.
- Sensitivity Analysis of features and hyperparameter of models with both good and poor accuracy.

The paper is arranged as follows: Section 2 presents the most promising ML-methods for PV power forecasting. Section 3 covers the methodology as: physics-informed data-mining, model tuning, evaluation and post-processing of results. Then, Section 4 applies the methodology to a study-case. Lastly, Section 5 highlights the key take-aways and possible future work.

## 2. Background

Different publications focused on solar forecast, have consistently identified ML as the most accurate and promising tools over the past few years given the growing data access capabilities. We introduce the most relevant in this section.

### 2.1. Random Forest

**Figure 1:**
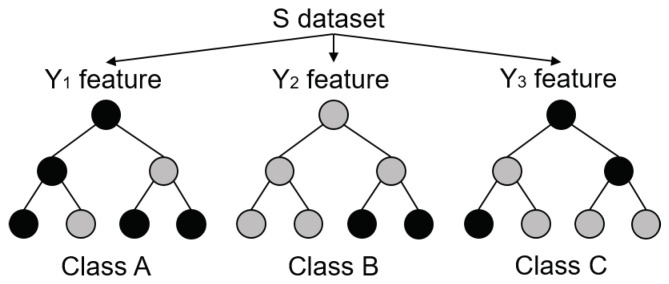
Simplified structure of a RF [25].

Decision trees are the simplest conditional decision making units. However, they tend to over-fit the data and present limited generalisation. Grouping them in the form of forest partially overcomes this limitation. Each tree in the forest draws a number of random samples with replacement increasing diversity as each tree is forced to use different data of the same size; leading to a more robust model. Each forecast is made as an average of the predictions of all the individual trees in the forest [23,24]. Despite, being originally developed for classification, RF are capable of performing time series regression, yielding excellent results in limited datasets with extremely noisy data [25]. Decision trees, depicted in Figure 1, split the data into subsets of homogeneous values according to simple if-then-else rules. Homogeneity is evaluated in terms of entropy as:

(1) $$E_{S}=\sum_{i=1}^{c}-p_{i}\log_{2}p_{i}\;\;\forall i\in \left[0,\;1,\;2,\;\ldots \;,c\right]$$

where $E_{S}$, $p_{i}$, and *c* stand for entropy of the training data at a given node, probability of class *i*, and number of classes. Subsequently, we can use Information Gain IG to check whether entropy can be reduced by splitting the data according to different *Y* subsets of the training dataset *S* as:

(2) IG(Y,S)=E(Y)−E(Y|S)

We then choose the subset with largest IG as decision node, creating a new branch. This is an iterative process that continues until the predefined depth has been reached.

### 2.2. Support Vector Machine

**Figure 2:**
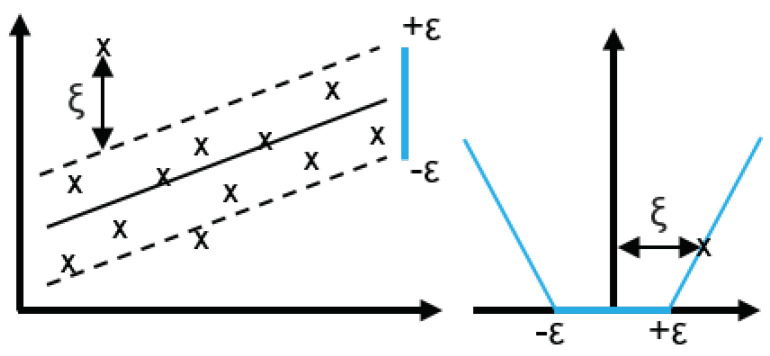
SVR basic concept [28].

Originally proposed by [26], SVM is a kernel-based supervised ML method originally developed for classification based on structural risk minimization of the training data. When applied to time series regression, it is known as Support Vector Regression (SVR). It maps the input data into a higher-dimensional space, then applies linear regression into that space; which is represented in Figure 2. The relevant hyperparameters to be tuned are three: penalty (ξ), radius (ϵ), and kernel function. The penalty determines the error weighting, radius fixes the data to be ignored, and kernel defines the optimization function [6,27].

### 2.3. Artificial Neural Networks

The architecture of any ANN consists of an input and output layers; with a variable number of hidden ones in between. Neurons conform the basic structure and represent the processing core of the model; while the information is exchanged between the different layers via synapses. Lastly, the activation function computes the prediction and forwards it as output. Specifically, LSTM belong to a subset of ANN called recurrent neural networks which are able to establish temporal sequences; making them best suited for time-series forecasting [21]. CNN, however, are used to discover relationships by filtering the available data [29]. Hybrid structures CNN–LSTM are currently the recommended approach when targeting day-ahead hourly resolution of both power and irradiance forecasting; as each sub-model can potentially capture spatial and temporal relationships, respectively, [30]. We have considered LSTM, CNN and hybrid architectures, which are gently introduced in this section [2].

#### 2.3.1. Long-Short Term Memory Neural Network

**Figure 3:**
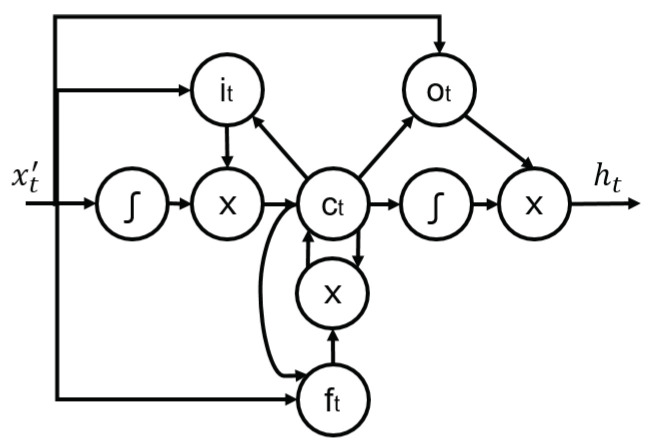
Neuron structure of an LSTM, reproduced from [31].

LSTM are designed to reduce the incidence of the vanishing gradient problem which causes ANN to stagger during training due to small convergence gradients in the optimisation solution. In LSTM, each cell-vector is capable of forgetting parts of their previously stored memory by adding new information. Figure 3 presents the LSTM neuron structure; note the lack of direct connection between input $i_{t}$ and output $o_{t}$. In this way, the information is forced through the memory unit or cell state $c_{t}$, as regulated by a memory preservation coefficient $f_{t}$ which is defined as in Equation (3). Where σ, $W_{fi}$, and $W_{fo}$, stand for the sigmoid gate, weights of the forget gate’s input and output tensor. While $x_{t}^{\prime }$, $h_{t-1}$, and $b_{f}$ represent the current state’s input tensor, previous state of the cell’s output and bias vector, respectively. Information is then transferred to $i_{t}$ and forwarded to $c_{t}$ as to save it in the long-term memory as in Equation (4).

(3) $$f_{t}=\sigma (W_{fi}\;x_{t}^{\prime }+W_{fo}\;h_{t-1}+b_{f})$$

(4) $$i_{t}=\sigma \left(W_{ii}\;x_{t}^{\prime }+W_{io}\;h_{t-1}+b_{i}\right)$$

where $W_{ii}$, $W_{io}$, $b_{i}$ stand for the weights of the input gate’s input and output tensor and the bias vector, respectively. Then, the previous cell state $c_{t-1}$ can be updated as Equation (5) leading to the neuron’s actual output as in Equation (6):

(5) $$c_{t}=\sigma \left(f_{t}\;c_{t-1}+i_{t}\right)$$

(6) $$o_{t}=\sigma \left(W_{oi}\;x_{t}^{\prime }+W_{oo}\;h_{t-1}+b_{o}\right)$$

where $W_{oi}$, $W_{oo}$, $b_{i}$ stand for the weights of the output gate’s input and output tensor and their related bias vector, respectively. The definitive output of the cell results:

(7) $$h_{t}=o_{t}\;\tanh\left(c_{t}\right)$$

LSTM algorithms should, in theory, excel in time series forecasting, since they capture temporal dependencies between observations [32]. A recent review on deep learning applications for renewable energy forecasting concluded that deep recurrent neural networks such as LSTM outperforms other traditional methods [9]. However, only for large enough datasets, since LSTM must train more parameters.

#### 2.3.2. Convolutional Neural Networks

**Figure 4:**
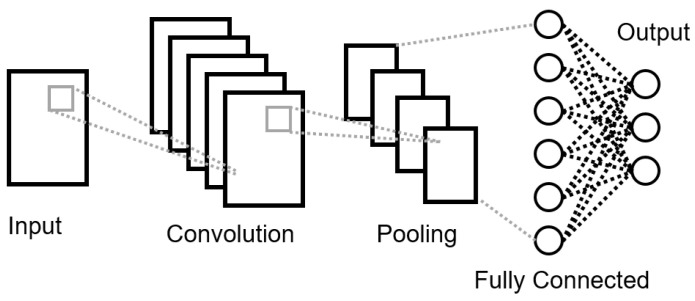
General Structure of an CNN.

CNNs are a type of ANN designed to resemble a grid as to accommodate data with spacial distribution. The term convolutional refers to integral blending operation or overlap between two functions. CNN uses this operation to extract inherent features from the previous layer. Whose type can be: input, convolutional, excitation, pooling, fully connected and output. Briefly, the input and output layers’ purpose is to retrieve and send the data, respectively, as depicted in Figure 4. Convolutional layers obtain local information from the data through different kernel activation functions. Passing a feature representation coming from previous layers trough user-defined activation functions producing an output feature map as in Equation (8). Then, the purpose of the excitation layer is to trigger the activation function increasing the non-linearity of the entire network. Pooling layers aim to reduce over-fitting by down-sampling the input data between successive convolution layers. There are different methods as described in [33], but no specific criteria related to which one to choose. Lastly, fully connected layers are used to predict a target variable based on the entire network, they are usually placed at the end of the structure. [34] CNN layers are stuck in different combinations to form increasingly complex structures. While neurons are not connected within, but across the same layer [33,35].

(8) $$h_{ij}^{k}=f\left({\left(W^{k}\;*x\right)}_{ij}+b_{k}\right)$$

where *f*, $W^{k}$, $k^{th}$, and ∗, respectively, stand for activation function, kernel’s weight, feature map, and convolutional operator.

#### 2.3.3. Hybrid CNN–LSTM

**Figure 5:**
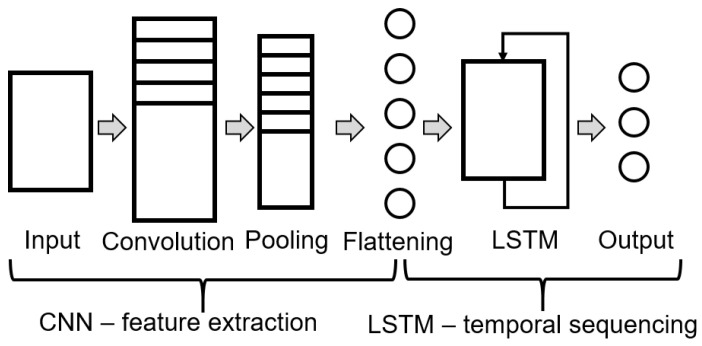
General structure of a hybrid CNN–LSTM ANN.

Recent publications point towards the combination of CNN and LSTM as a hybrid model. This concept extracts spatial features with CNN and temporal with LSTM. Authors do not agree on using CNN at the top of the architecture as a data filter and leave LSTM at the core; or vice versa. Figure 5 presents the hybrid CNN–LSTM structure [30,34].

## 3. Methodology

**Figure 6:**
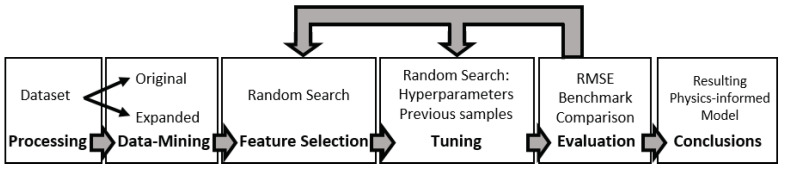
Methodology flow.

Obtaining generalized results is an inherent challenge of ML-methods particularly for RES-based applications since the datasets are usually limited to a single location or climatic zone. To overcome this challenge, we have designed a methodology that, as depicted in Figure 6, first pre-processes and expand a basic dataset with physics informed metrics. Then, it selects features and tunes hyperparameters in a concurrent and iterative manner, subsequently finding common patterns in the best performers of each model. In this way, we are able to survey the whole search space and narrow down the configuration of the best performer to a small search space. Lastly, the resulting models are evaluated, leading to generalisations in the behavior of the ML-models. This section covers the steps followed in such assessment, which can be easily replicated by the interested reader using [36].

### 3.1. Physics Informed Data-Mining

Physics informed ML is currently a rapidly evolving field whose main goal is to introduce knowledge from the real world physical dependencies in the ML models. This is done to enrich the available dataset with background knowledge of the forecasting field. In order to generate physics informed features and introduce them into the different ML-models, we used a classic PV performance representation called “Power-temperature coefficient model” given its good accuracy and simplicity; and since it is possible to build using the PV panel’s data-sheet and measurements of wind speed and ambient temperature. Yet again, neither those conditions is generally problematic given the widespread data access [37]. The Plane Of Array irradiance or $E_{\mathrm{POA}}$ [W/m${}^{2}$] corresponds to the total solar irradiance incident on a PV panel’s. It depends on the sun’s position, the array’s orientation, the reflectivity of the surroundings and shading. It can be computed with Equation (9); where $E_{b}$ and $E_{d}$ stand for beam and diffuse irradiance, while $E_{g}$ does so for the irradiance reflected on the surroundings. Since the array understudy is fixed with the same orientation and inclination as the tilted irradiance sensor, we can consider those recordings to capture $E_{\mathrm{POA}}$ directly.

(9) $$E_{\mathrm{POA}}=E_{b}+E_{d}+E_{g}$$

Then, the temperature in [K] of the module ($T_{m}$) can be estimated with Equation (10). Where $T_{a}$ stands for ambient temperature, *a* and *b* represent two coefficients related to module material and construction parameters as according to [37]. These and other parameters related to the specific PV panels used in this study are presented in Table 1. Furthermore, cell temperature Tc is computed as defined in Equation (11); where $E_{\mathrm{STC}}$ represents the reference irradiance (1000 W/m${}^{2}$) and ΔT is a data-sheet parameter representing the difference between the module and cell temperatures.

(10) $$T_{m}=T_{a}+E_{\mathrm{POA}}\;\exp\left(a+b\;W_{s}\right)$$

(11) $$T_{c}=T_{m}+\frac{E_{\mathrm{POA}}}{E_{\mathrm{STC}}}\Delta T$$

Subsequently, the DC output power of the PV panel ($P_{DC,panel}$) can be computed using Equation (12); where $P_{mp,STC}$, $T_{STC}$, and $\gamma_{mp}$ stand for the panel’s peak power [W] and temperature measured under standard conditions, and the normalized temperature coefficient of peak power, respectively. The output power of the array can be obtained then, by multiplying $P_{mp,panel}$ with the number of panels connected in series and in parallel as in Equation (13). Finally, the output power of the array is obtained with Equation (14) by applying the inverter’s efficiency as stated in its datasheet. Thus, obtaining the estimated power output of the array, that is, $P_{\mathrm{AC}}$ in [W]. This value is used to identify curtailment periods and inaccurate active power measurements simply by locating large deviations (more than 50%) and substituting the wrong value with $P_{\mathrm{AC}}$. The number of substituted values corresponds to less than 0,5% and were concentrated consecutively during short periods of time (that is a couple of hours due to maintenance, running experiments, etc).

(12) $$P_{DC,panel}=\frac{E_{\mathrm{POA}}}{E_{\mathrm{STC}}}P_{mp,STC}\left[1+\gamma_{mp}\left(T_{c}-T_{STC}\right)\right]$$

(13) $$P_{DC}=P_{DC,panel}\;N_{s}\;N_{p}$$

(14) $$P_{\mathrm{AC}}\left\{\begin{array}{cc} \eta_{max}\;P_{DC} & if\;\eta_{max}\;P_{DC}\leq \;P_{ACmax}\\ P_{ACmax} & if\;\eta_{max}\;P_{DC}>\;P_{ACmax} \end{array}\right.$$

Introducing this model as part of any ML-model allows to effectively integrate physical knowledge in the forecaster. Theoretically, given enough iterations, ML should be capable by itself to learn these relationships. Specially if we were to feed them as part of different models and then casting a prediction as an ensemble. However, by computing them ourselves in advance we speed up the learning process, reduce memory needs and improve accuracy. In addition, we decided to include a metric covering the time of the day as the number of hours from 0 to 23, ($H_{\mathrm{day}}$). Intuitively, we understand that, by introducing the time of the day in the model, we get an idea of the potential production and the day/night cycle.

### 3.2. Model Tuning

The three most important steps in ML are feature selection, number of previous samples, and hyperparameter tuning. One of the main problems is that they cannot be addressed independently, since two models trained with either distinct features, number of previous samples or hyperparameter configuration are effectively different. Thus, their selection and tuning must be addressed simultaneously. Nevertheless, this topic has received extensive attention in the scientific literature. Intuitively we can understand that, by choosing the wrong features, a model can not learn anything valuable and, thus, will be unable to predict anything. Yet, in general, publications from computer science related journals would be a bit inflexible regarding this question, while other more application oriented recommend to address feature selection first; or at least to limit the number of features to a minimum [38,39]. Hence, this approach constitutes the general tendency in the vast majority of published work focused on both irradiance and PV power forecasting. Briefly, feature selection can be addressed in either an unsupervised or supervised fashion. The first rely solely on the data by focusing on model-independent techniques such as Pearson correlation. This approach is both naive and powerful as it focuses on a particular type of relationship between the different metrics, linearity; while leaving out others. Normally, it provides a good approximation even if it does not configure the optimal model. On the other hand, supervised feature selection seeks to find the best set of input features maximizing a model performance. However, this implies that this selection is model-dependent and what it might be the best combination for one model is not necessarily true for another. Thus, introducing a bias in the analysis. A fairly common technique covering simultaneous feature selection and tuning is the so-called brute force approach. Which basically aims to test all possible combinations of features and hyperparameters. The main problem of this approach is the extremely large requirements in both computational capacity and time. Yet, the previously discussed methods can be used to reduce the search space for this brute-force approach. This is sometimes referred to as grid-search method.

### 3.3. Feature Selection

Approaching feature selection with a simple comparison in terms of Pearson correlation constitutes the common practice. Defined in Equation (15), this metric expresses the linear relationship between two variables with values ranging from −1 (fully anti-correlated) to 1 (fully correlated); while 0 means that there is no linear relation whatsoever. Indeed, values with large Pearson values tend to be generally useful for any ML-method since linearity marks a strong relationship between features. However, the opposite statement is not necessarily true. Most papers point out the ability of ML-methods to learn from non-linear features, particularly of ANN, and yet these are intentionally not included [5]. One reason for this is the non-existence of an unique metric of non-linear dependence; as it can only be assessed for particular relationships, e.g., polynomial logarithmic, etc.

(15) $$R_{X,Y}=\frac{N\sum XY-\sum X\sum Y}{\sqrt{N\sum X^{2}-{\left(\sum X\right)}^{2}}\sqrt{N\sum Y^{2}-{\left(\sum Y\right)}^{2}}}$$

**Figure 7:**
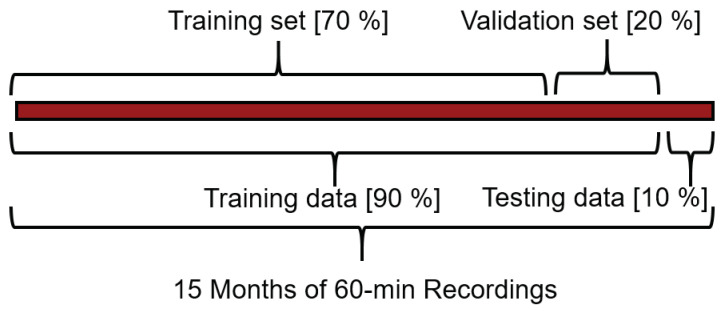
Data division approach.

In addition, the information provided by two or more features might give redundant results. For example, $P_{\mathrm{DC}}$, $P_{\mathrm{AC}}$, $T_{m}$, and $T_{c}$ as defined in Equations (10)–(14) provide an estimate of the power and temperature, respectively. The differences are minor, $P_{\mathrm{AC}}$ includes the efficiency of the inverter, while $P_{\mathrm{DC}}$, does not; then $T_{m}$ is related to the module and $T_{c}$ to an individual cell. Intuitively, we understand that, even though they contain different information, this is closely related, and redundant up to some point. Furthermore, we are using $T_{a}$, $W_{s}$ and irradiance to compute $T_{m}$, and $T_{c}$. The first three are related to weather conditions, while the last two are directly coupled to the PV’s performance, thus, we expect them to show higher relevance. This allows to remove the meteorological factors in favour of the newly computed metrics. In this way, the model’s complexity is kept in check without information loss. Lastly, other metrics might hold non-linear relationships such as the time of the day, which will not present a high Pearson value, and yet, we are physically aware of its relevance. The classical unsupervised approach based solely on Pearson fails to identify both concepts. Hence, we complement that classic approach with physics-informed decisions. Now, for any practical forecaster development, it is necessary to iteratively train a number of models with different combinations of features and hyperparamters. This process follows a grid search aiming to cover as many combinations as possible. The whole purpose of the unsupervised feature selection is to reduce this search-space. However, in order to demonstrate the usefulness of a physics informed selection we considered all of the available features in different combinations with a limited number of hyperparameter configurations and number of previous samples. The data of the selected features is divided into different subsets for training and testing as according to Figure 7, note that the subsets’ proportion is chosen as common practice from general ML applications on forecasting. Then, certain accuracy or error metrics are used to rank the different models.

### 3.4. Tuning and Evaluation

Once the set of feature combinations is defined, we train them with a random configuration of previous samples and hyperparameters. The resulting models are evaluated on the testing set according to two metrics, Mean Absolute Error (MAE) and Root Mean Square Error (RMSE) as defined in Equations (16) and (17). MAE evaluates the uniformity of the forecast errors by equally weighting all the errors due to its averaging process, thus representing an estimate of the median. Similarly, RMSE gives the best estimate of the conditional expected value by focusing on the overall model accuracy since it magnifies undesirable large deviations. Neither method is perfect, but their combination results in a solid analysis. We use MAE as the training objective, while, RMSE is employed to evaluate accuracy on the testing dataset. Then, model ranking is established using the average RMSE over the forecasting horizon ($\bar{\mathrm{RMSE}}$).

(16) $$MAE=\frac{\sum_{i}^{n}\left|Observation_{i}-Prediction_{i}\right|}{n}$$

(17) $$RMSE=\sqrt{\frac{\sum_{i}^{n}{\left(Observation_{i}-Prediction_{i}\right)}^{2}}{n}}$$

In addition to comparing the selected ML-models among themselves, we use Persistence and SPAR as performance benchmarks. The former assumes the state of a dynamic system at a particular time index *t* to continue fixed for another instant t+δt. Typically, fixing the last available measurement for the upcoming prediction horizon as expressed in Equation (18), where P stands for active power production measured in the system understudy and H represents the prediction horizon [1].

(18) $$P_{\left(t+\Delta t|t\right)}=P_{t}\;\;\forall \;\;\Delta t\;\;\in \;\;H$$

Semi-parametric modeling techniques have been extensively employed in recent decades in statistical modeling [40]. SPAR models include a diurnal curve intercept and an auto-regresive term using spline functions (fbs()) based on the time of the day (tday) as:

(19) $${\hat{P}}_{\left(t+\Delta t|t\right)}=f_{\mathrm{bs}}\left(t_{\mathrm{day}}\right)+f_{\mathrm{bs}}\left(t_{\mathrm{day}}\right)P_{t}\;\;\forall \;\;\Delta t\;\;\in \;\;H$$

SPAR fits the data by firstly calculating the base splines, resulting in 20 separate time-series with 10 degreess of freedom. Then a regression is carried out with a recursive least squares scheme; ultimately achieving coefficients able to adapt to data over time [8]. Such procedure only uses the latest power observation and is repeated for each horizon.

### 3.5. Post-Processing

We recorded the number of times each feature appears in a model able to beat Persistence for all the samples over the considered horizon. Then, we can evaluate a feature’s importance as the normalized number of times it appears as part of a model beating the benchmark for a particular resolution and horizon in any combination of features, ML-model, hyperparameters and number of previous samples. However, sufficient tuning diversity must be ensured to avoid biases. This problem is smoothed out simply by introducing a large number of iterations with random selection. The features appearing the least number of times can be discarded from the overall dataset, thus reducing the search-space. On the other hand, the remaining features are used to define a number of possible feature combinations. In addition, $\bar{\mathrm{RMSE}}$ is then used to elaborate rankings related to which ML-model returns the lowest error with which feature combination and number of previous samples. This approach, if using a sufficiently large number of iterations as to ensure statistical significance, can be used to generalise regarding what are the most significant features, ML-model and number of previous samples. Such conclusions can then be used to further tighten the search space. Then, we find the best performers for each ML-method and compare them among them. Nevertheless, it should be noted that the proposed approach is meant to allow for generalization regarding feature selection. A practical development of a PV-forecaster would simply select the physically relevant features and proceed to hyperparameter tuning. We will now show a practical implementation of this methodology for a particular site.

## 4. Study-Case

A PV installation located in Roskilde, Denmark is used to demonstrate the proposed physics-informed feature selection. We considered an hourly sampling rate and three different horizons: 24, 48 and 72 steps ahead. The importance of this horizon/resolution combination is related to their use in day-ahead energy markets and other energy management applications such as the scheduling of energy storage, flexible loads and energy market participation [22]. Complementarily, a relatively low resolution such as hourly, allows to train models quite fast.

### 4.1. Data-Description

We employed data from SYSLAB, a laboratory for distributed energy resources part of the Danish Technical University (DTU) located in Risø, Denmark. The set-up includes a meteorological mast and a 10 kW PV array whose data is presented in Table 1. Both devices transmit their recordings to a central data logger via TCP/IP communication with a resolution of 1 second which is then averaged over one hour. The PV inverter records active power on the AC side, while the meteorological mast measures GHI, Plane of Array irradiance ($E_{\mathrm{POA}}$), wind speed ($W_{s}$), wind direction ($W_{\mathrm{dir}}$), air temperature ($T_{a}$) and humidity. All metrics are recorded with a 1% error, except for humidity and irradiance which present 0.6% and 3% respectively. The PVs are tilted 60° and are partially shaded by a nearby wind turbine, bushes, trees and buildings. The mast is approximately 10 meters apart, thus some spatial correlations might not be captured, for example fast moving clouds. Since the PV system is installed in a research facility, it is sometimes used curtailed as a dispachable unit. Therefore, the recorded active power does not necessarily resemble the available solar power. However, this limitation is solved during data-preprocessing. We used 15 months of recordings from the first of June 2019 to the 31 August 2020 which are made available to the reader at [41].

### 4.2. Preprocessing and Data-Mining

The first step in the data preprocessing is to deal with the timestamp misalignment. This is a fairly common issue in systems with asynchronous recording. The approach was simply to generate all the possible timestamps for the considered period in hourly samples, then to fill them with the data available while the rest with NAN values. Subsequently, the dataset was expanded with the physics-informed metrics as described in Section 3.1. Regarding missing data, if the value corresponded to a power measurement, it was substituted with the computed $P_{\mathrm{AC}}$ if available. Otherwise it was kept as a NAN value. Horizons containing NANs after the cleaning process were dropped from the dataset, which accounted for less than 0.1% of the dataset. Then, the data was divided into sets for training, validation and testing as according to Figure 7. Subsequently, the sets were scaled from 0 to 1 as according to the values of the training set to avoid cross-contamination.

### 4.3. Feature Analysis

**Figure 8:**
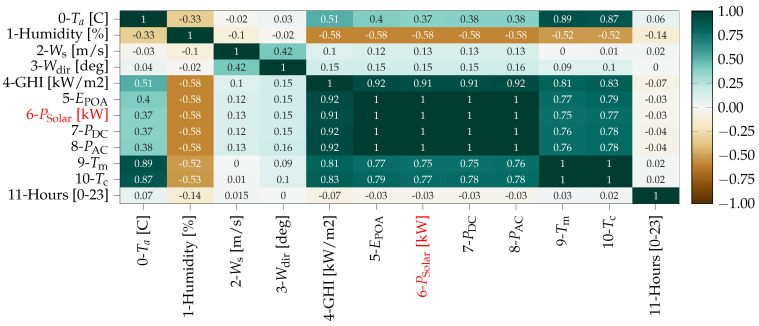
Heatmap representing Pearson correlation.

Figure 8 represents the Pearson correlation for the complete dataset, note that the variable representing the intrinsic variable, is highlighted in red. Furthermore, the original set is conformed with features 0 to 6 while 7 to 11 does so for the expansion. As expected, $E_{POA}$, $P_{\mathrm{DC}}$ and $P_{\mathrm{AC}}$ show perfect linear correlation with the intrinsic feature. More interesting though is the fact that $T_{a}$ and specially $W_{s}$ present very low values. However, after combining them with Equations (10) and (11); which are clearly non-linear, the resulting $T_{m}$ and $T_{c}$ also present high correlation values. Regarding the time of the day, $H_{\mathrm{day}}$ presents near 0 correlation, thus being discarded from the suitable features according to Pearson’s criteria. Lastly, humidity exhibits a relatively high anti-correlation. Then, according to the Pearson approach the selected features would be: humidity, GHI, $E_{\mathrm{POA}}$, $P_{\mathrm{DC}}$, $P_{\mathrm{AC}}$, $T_{m}$, and $T_{c}$. However, for the physics-informed selection method, we eliminate redundancies first and focus on the most physically relevant metrics. For instance, $E_{\mathrm{POA}}$ captures the irradiance hitting the PVs while GHI is more of a general measurement. $P_{\mathrm{AC}}$ includes also the effect of the inverter’s efficiency and $T_{c}$ represents better the thermodynamical state of the panel. In addition, humidity includes information related to the heat transfer from the PV to the environment and the possibility of rain. Lastly, $H_{\mathrm{day}}$ is a clear marker of the daily solar cycle. Hence, the Proposed physics informed approach selects: humidity, $E_{\mathrm{POA}}$, $P_{\mathrm{AC}}$, $T_{c}$, and $H_{\mathrm{day}}$.

### 4.4. Training and Evaluation

Considering that any set with *n* elements has exactly $2^{n}$ subsets, for the given dataset, there are 2048 feature combinations. During the practical development of a forecasting tool, we would be able to tight the search-space to the Pearson or the Physics-informed which would reduce the number of sets to 256 and 64, respectively. However for the purpose of generalising the findings of this work, we considered a wide spectrum of hyperparameters and number of previous samples combined with all the features and conducted a random search. In that sense, we consider that the number of previous values can be selected from 0 to 96 in steps of 6. Then, regarding hyperparameter tuning, RF can take from 100 to 1000 trees in steps of 100, while SVR could use rbf, polynomial (from 1 to 5 degrees) and sigmoid kernels, with the C parameter as an integer from 1 to 7 in steps of 2, epsilon from 0.1 to 1 in 0.1 steps. Regarding ANN in general, we found that batch sizes of 16 were the most convenient along with epocs of about 1000. Particularly for CNN, it could take from 12 to 60 filters in steps of 12, kernel and pooling sizes as integers from 1 to 5. Then, for LSTM, in the first run we allowed structures from 1 to 3 layers with 5 to 20 neurons/layer in steps of 5. Finally, the hybrid CNN–LSTM could chose the combined possibilities of CNN and LSTM. Note that the same configurations are applied to the three selected horizons of 24, 48 and 72 h ahead. Accuracy is evaluated in terms of RMSE over the testing set for all the models omitting night periods as to avoid unrealistic good results. These periods are identified with two conditions, first a measured production below 10% and second, time of the day outside of 7 to 19 h interval.

### 4.5. Discussion of Results

**Figure 9:**
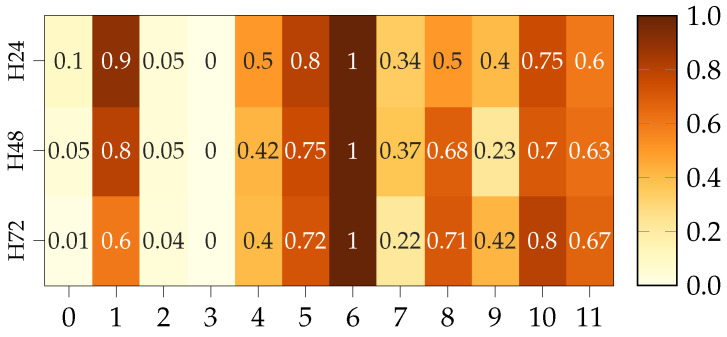
Heatmap features top performance.

Figure 9 presents the normalized number of times a particular feature appears as part of a model able to beat persistence in terms of $\bar{\mathrm{RMSE}}$. Note that features are identified with the ordinal number from Figure 8. The more a feature appears in the most accurate models, the more relevance or learning potential it holds. In the image, we can see how the features repeating more often correspond to those belonging to both Pearson and the Physics-informed method, which confirms the unsupervised feature selection criteria. It should be noted how these results are consistent among the different horizons, pointing towards the idea that the group of useful features is quite consistent irrespective of the particular horizon. Similarly, $\bar{\mathrm{RMSE}}$ was used to evaluate which feature combination and number of previous samples return the lowest errors for each model.

**Figure 10:**
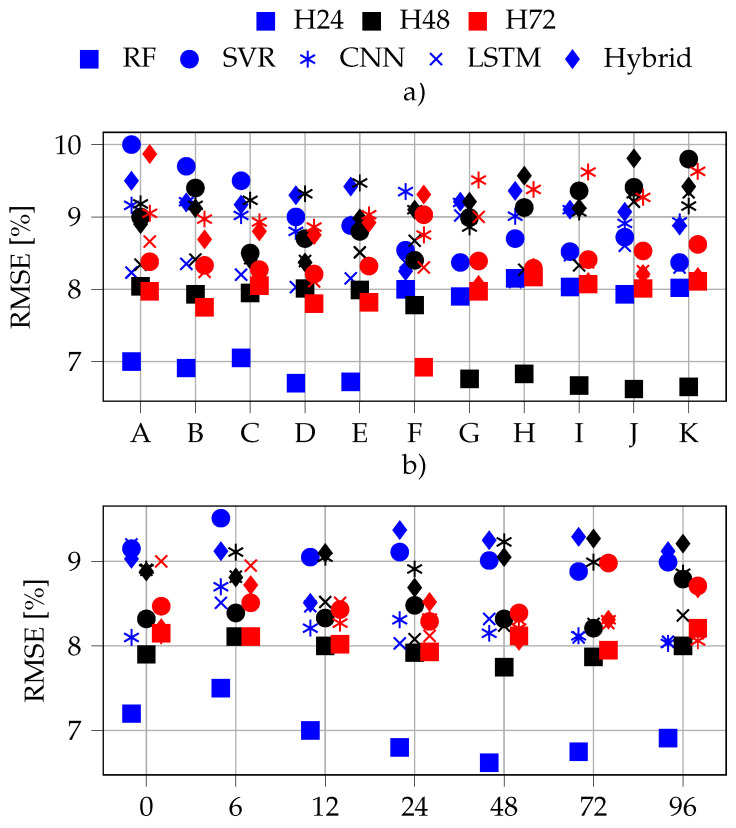
Top combinations: features (**a**); previous samples (**b**).

The top 10 feature combinations are presented in Figure 10a, note that the list of combinations is summarized in Table 2; while Figure 10b focuses similarly on previous samples. Note that, the combinations on the y-axis are those of the best performer for each configuration, that we present the three horizons and 5 ML-methods at once. In that sense, the x-axis must be taken as a category and not a scale. H24 and H72 have a negative and positive offset, respectively, as to facilitate visualization of the three horizons at once. Regarding the consistency of this method, there are a number of interesting conclusions to be dragged from these images. Some are quite intuitive, such as that the $\bar{\mathrm{RMSE}}$ is directly proportional to the horizon length for each model independently, this can be seen in both pictures. Then, all the top 10 feature combinations belong to the physics-informed feature selection. Thus, this method effectively reduces the search space while still capturing the most important ones. Furthermore, there is a threshold for the number of previous samples to be included, for example in the case of RF, its optimal point is placed between 24 and 72, possibly in the vicinity of 48, as we can see how the error minimizes around that point in Figure 10. This threshold is however different between horizons except for CNN and LSTM which seem consistent. Error distribution is consistent for a set of ML-model and horizon, meaning that, the accuracy of the best model for a particular ML falls within a relatively narrow band disregarding number of previous samples and feature combination, as long as the latter is build following the physics informed approach. This is extremely meaningful, as it ensures that, as long as the feature combination is taken from the physics-informed subset, it will very likely lead to a top-performer. Therefore, the focus is to be put on the hyperparameters tuning.

In general, both figures show how CNN–LSTM and CNN rank as the two worst models in all the horizons, while RF and LSTM lead in accuracy on the 24H. Then, RF dominates in both H48 and H72, where SVR and LSTM return similar performances. Regarding feature combinations, B, D, I and J return the overall lowest error for all the models. Note that this combinations basically use humidity, $E_{\mathrm{POA}}$, $T_{c}$ and $H_{\mathrm{day}}$. Lastly, $P_{\mathrm{AC}}$ is the feature least repeated in the best performers, the reason could be that it might result a bit redundant comparatively with the rest since it is calculated as a combination of them. Nevertheless, the most important point is that none of the features not included in the physics-informed feature set made it to the top performers for any of the ML-methods.

### 4.6. Best Performing Models

**Figure 11:**
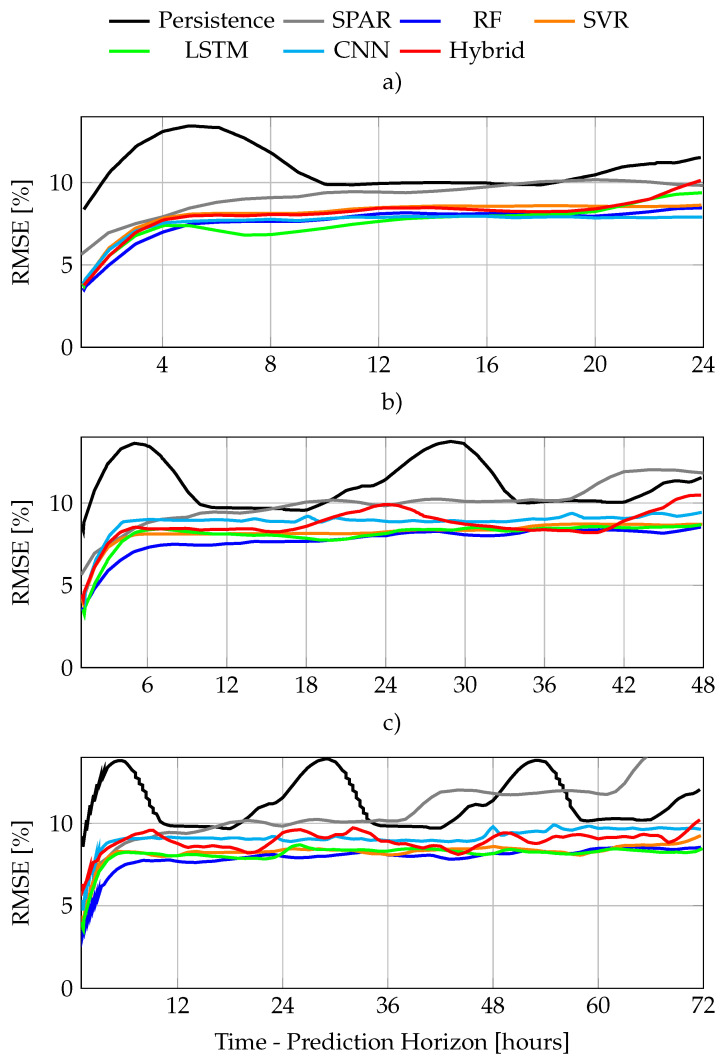
RMSE comparison for differet horizons: (**a**) one, (**b**) two, and (**c**) three days ahead, respectively.

Figure 11a, b and c present the RMSE of the best performers of each ML-method for the three different prediction horizons, 24, 48 and 72 h, respectively. Then, Table 3 and Table 4 present the hyperparameters and feature configuration. Note how, again, features are identified with numbers matching those of Figure 8. While Table 3 also presents the ranking of the models for each horizon in terms of $\bar{\mathrm{RMSE}}$.

**Figure 12:**
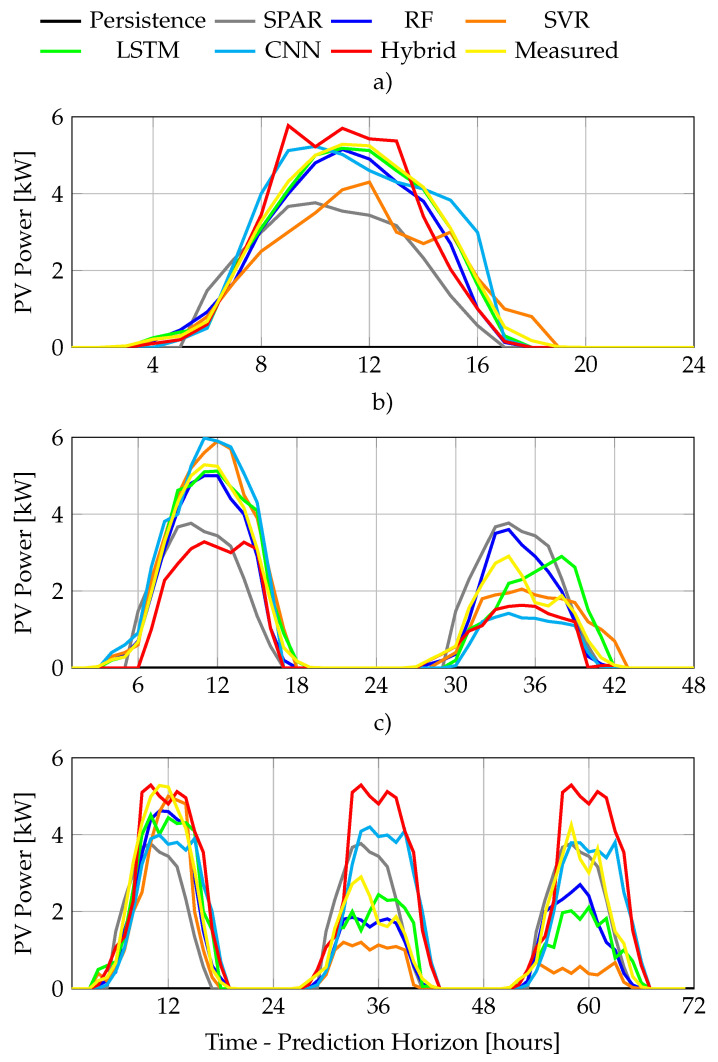
Sample forecast comparison: (**a**) one, (**b**) two, and (**c**) three days ahead, respectively.

In general terms, Figure 11 shows how RF presents an almost linear decay with the increasing horizon, while SVR’s performance is slightly worse, but decays at a lower rate. On the other hand, all the ANN present a fast performance decay beyond 24 h, that reduces the decay tendency afterwards. As shown in Figure 11a, LSTM is the best performer for the 24H while RF dominates Figure 11b,c. SVR is third in Figure 11a,b, but second in Figure 11c as it decays more slowly than LSTM. CNN–LSTM and CNN consistently ranked at the bottom of the list for all the horizons. A forecast example for the days 3 to 6th of August as 24, 48 and 72 h ahead are presented in Figure 12a–c, respectively.

It is fairly difficult to compare results across different publications without falling into naive observations pointing towards the better performance of the proposed method. Factors affecting accuracy besides the prediction method are: the predicted metric (as most work forecast irradiance and estimate power), horizon, sampling rate, dataset length, geographical location, period of the year, and array size to name a few. Therefore, it is impossible to perform a trustworthy comparison without having access to the full dataset, including enough information to replicate our methodology. This is the main reason behind sharing our original dataset and scripts, to facilitate the replicability of our work.

The previous literature highlighted the ability of deep-learning methods such as CNN, LSTM, and hybrid to return hourly day-ahead forecasts with errors ranging from 8 to 15% using NWP [42,43,44]. In this work, we are able to obtain an error with 7.5% RMSE on average with LSTM for the 24 h horizon (day-ahead) without the need for NWP. In fact, the results presented in Table 3 show how the best performers improve those results. We included these values to point towards the suitability of our method. Nevertheless, they should be taken very cautiously.

For H24, LSTM was trained with a combination of 96 previous samples, including humidity, $E_{\mathrm{POA}}$, active power and $T_{c}$. This is clearly a fairly complex model that seems to deteriorate very fast after the 20 h horizon. Then, for H48, RF presents the lowest error with 7.7% RMSE on average. Particularly a combination of 350 trees, Humidity and 48 previous samples of measured active power. In general, for this horizon, humidity and $E_{\mathrm{POA}}$ appear as the most useful features. However, a LSTM including the hour of the day and 24 previous samples manages to beat RF for a few samples. Lastly, for the 72 h horizon, RF including 350 trees, humidity and 24 previous samples of measured power presents the best performance with 7.9% RMSE on average. Note that the only difference with the 48 h horizon is the number of previous samples. The closest competitors used either higher number of previous samples, $P_{\mathrm{AC}}$ or $T_{c}$. One simple explanation for RF representing the best performer for both 48 and 72 h-ahead is the highly volatile Danish weather; which difficulties finding patterns based on deep learning. The results could change for shorter horizons, or larger dataset spawning several years, in this way, ANN could potentially learn how to capture this inherent weather randomness. For the employed dataset, volatility can be understood as data noisiness, which RF captures very effectively. Nevertheless, ANNs are expected to be the best performers where predicting one or very few steps ahead, where temporal correlations hold more relevance.

Following the recommendation in [22], we can discuss some of the findings regarding under-performing methods. Regarding hyperparameter tuning, RF above 500 trees systematically under-performed for the same feature combination. The same can be stated about SVM with C values above 3, ANN deeper than 3 layers or including dense ones (which could be explained by under-fitting due to insufficient samples). Regarding the Hybrid method, some authors recommended 2-D convolutions, however, we found 1-D ones yielded more accurate results. Regarding feature sensitivity, intrinsic models generally yielded acceptable accuracy, extrinsic feature inclusion did not always improved the performance, unless chosen from the physics-informed metrics. The results can be summarized as:

1. The proposed features based on conventional PV performance models have proven their usefulness. Particularly, non-redundant features: $E_{\mathrm{POA}}$, $P_{\mathrm{AC}}$, $T_{c}$, and $H_{\mathrm{day}}$, are part of the most useful features on the three horizons. Thus, we recommend their inclusion in future studies focused on data-driven PV power forecasting.
2. Unsupervised feature selection based on correlation is a naive approach not suitable for physics-informed models. Accordingly, the selected features should be GHI, $E_{\mathrm{POA}}$, $P_{\mathrm{AC}}$, $P_{\mathrm{DC}}$, $T_{m}$, $T_{c}$. Humidity and $H_{\mathrm{day}}$, would be discarded despite being part of the best performers of each horizon. We can not consider this to be a coincidence given the large number of trained models and the result consistency. Furthermore, several metrics can present high values of linear correlation and not include meaningful potential learning as it captures the same phenomena. That is for example the case between GHI and $E_{\mathrm{POA}}$, $P_{\mathrm{DC}}$ and $P_{\mathrm{AC}}$, $T_{m}$ and $T_{c}$; including either one is useful, but both add more complexity than learning to the model. Therefore, physics-informed feature selection is preferred as it includes the best performers while representing a 75% smaller search-space than Pearson-based; which does not include all the best.
3. In general, the best feature combinations were performing good for all the different methods. The differences among the top 10 were about 0.5% RMSE difference.
4. Deep-learning is the method to use for day-ahead prediction, but not for longer horizons as its accuracy deteriorates fast above 20 h ahead due to weather volatility. Other ensemble methods such as RF are better suited for pattern recognition of noisy data under limited datasets. However, this could be overcome by including NWP.

## 5. Conclusions

This paper proposed improving ML-based PV power forecasters by extending a given dataset with PV-performance models and other physically relevant variables. These models learn about the physical relationships between an PV power and environment; potentially leading to more accurate models than conventional methods. Given the difficulties in proving generalizations on the ML field, we developed a methodology providing statistical relevance of our claims and then applied it to a study case.

Briefly, after expanding the dataset, a random search is conducted given a sufficiently large set of possible hyperparameter and previous samples configurations. The models’ whose $\bar{\mathrm{RMSE}}$ outperform two benchmarks are deemed useful and their features recorded. The most repeated features were consistent among models and horizons, corresponding to the physics-informed search-space.Then, the 10 best feature combinations and number of previous samples were selected based on $\bar{\mathrm{RMSE}}$ to show performance uniformity among the different ML-models. There, we could see how each model presents similar accuracy within a relatively narrow bandwidth, while there were substantial differences between methods and horizons. In addition, the optimal number of previous samples could also be estimated from this analysis as there was a clear convex tendency. Lastly, we presented a performance comparison of each ML-model, Persistence, and SPAR for each horizon. The best performers use features from the physics-informed set, thus the proposed dataset expansion is useful in improving the accuracy of a ML-based PV power forecasting tool.

Among the advantages of the proposed method are, first, the tightening of the physically informed search space, effectively easing training. Second, its suitability for isolated or autonomous systems. Third, its simplicity and applicability to virtually any PV plant as it is based on basic meteorological data and datasheet values. On the other hand, household level installations (prosumers) might not have meteorological stations close enough to provide the necessary data. Similarly, NWP or sky imaging were not available.

In the study case, RF and LSTM presented the best accuracy, however, this results could vary for other horizons or after including NWP. Future research could focus on studying the suitability of this method for higher sampling rates, as some of the proposed metrics may not work well given their slow thermodynamical behavior.

## Figures and Tables

**Table 1:** PV system data.

Parameter	Value
a	−3.56
b	−0.075
ΔT	3
$P_{\mathrm{mp},\mathrm{STC}}$	200 W
$\gamma_{\mathrm{mp}}$	−0.00478
$N_{s}$	18
$N_{p}$	2
$P_{\mathrm{ACmax}}$	10 kW
PV manufacturer	Schüco
Inverter	SMA SunnyTripower 10000TL

**Table 2:** Top feature combinations.

ID	Features	ID	Features
A	6		
B	1, 6	G	5, 6, 10
C	6, 11	H	6, 9, 11
D	1, 5, 6	I	1, 5, 6, 11
E	1, 6, 8	J	1, 5, 6, 11
F	1, 6, 10	K	6, 7, 9, 11

**Table 3:** Best ML-method hyperparameter tunning.

Hor	RF	SVR			LSTM			CNN					Hybrid					
	Trees	Kernel	C	ϵ	NN	b	ep	fil	k	p	b	ep	NN	fil	k	p	b	ep
24	500	rbf	3	0.1	15-5-10	16	500	16	3	3	16	100	15-10	32	3	2	16	1000
48	200	rbf	2	0.1	10-15	16	1000	32	2	3	16	100	15-15-15	32	3	2	16	1000
72	350	rbf	3	0.1	15-10	16	500	32	3	3	16	100	15-10	32	3	2	16	1000

**Table 4:** Best ML-method Configuration & Results.

Hor	RF			SVR			LSTM			CNN			Hybrid		
	Pre	Feat	$\bar{\mathrm{RMSE}}$	Pre	Feat	$\bar{\mathrm{RMSE}}$	Pre	Feat	$\bar{\mathrm{RMSE}}$	Pre	Feat	$\bar{\mathrm{RMSE}}$	Pre	Feat	$\bar{\mathrm{RMSE}}$
24	48	1, 5, 6, 12	7.58	72	1, 6, 8	8.06	24	1, 5, 6	7.56	96	1, 6, 10	8.69	96	1, 5, 6, 10	8.06
48	48	1, 6	7.75	72	1, 5, 6	8.21	24	6, 11	8.08	96	1, 5, 6	8.86	24	1, 6	8.69
72	24	1, 6	7.93	24	6, 8, 12	8.29	24	7, 8, 12	8.12	96	6, 8, 12	9.16	48	5, 6, 10	8.96

## Data Availability

In order to increase transparency and the replicability of the work we share the employed dataset in a repository at DTU DATA [41]. Furthermore, we also share several Python scripts based on Open Access libraries with two purposes. First, to facility the access to the dataset and, second, provide an easy to use platform applying the physics informed methodology discussed in this work. The Python scripts are available at [36].
